# Identification of Tumor Antigens in the HLA Peptidome of Patient-derived Xenograft Tumors in Mouse

**DOI:** 10.1074/mcp.RA119.001876

**Published:** 2020-11-23

**Authors:** Nataly Mancette Rijensky, Netta R. Blondheim Shraga, Eilon Barnea, Nir Peled, Eli Rosenbaum, Aron Popovtzer, Solomon M. Stemmer, Alejandro Livoff, Mark Shlapobersky, Neta Moskovits, Dafna Perry, Eitan Rubin, Itzhak Haviv, Arie Admon

**Affiliations:** 1 Department of Biology, Technion–Israel Institute of Technology Haifa, Israel; 2 The Azrieli Faculty of Medicine, Bar Ilan University, Safed, Israel; 3 Institute of Oncology, Davidoff Center, Rabin Medical Center and Sackler Faculty of Medicine, Tel-Aviv University, Petah Tikva, Israel; 4 Davidoff Center, Rabin Medical Center, Beilinson Campus, Petach Tikva, and Felsentien medical research center, Petach Tikva, and Sackler Faculty of Medicine, Tel Aviv University, Tel Aviv, Israel; 5 Institute of Pathology, Barzilai University Medical Center, Ashkelon, Israel; 6 Davidoff Center, Rabin Medical Center, Beilinson Campus, Petach Tikva, and Felsentien medical research center, Petach Tikva, Israel; 7 Faculty of Health Sciences, Ben-Gurion University of the Negev, Beersheva, Israel; 8 The Shraga Segal Department of Microbiology, Immunology and Genetics, Ben-Gurion University of the Negev, Beersheba, Israel

**Keywords:** Mass spectrometry, cancer biomarker(s), peptides, immunology, clinical proteomics, peptidomics, personalized medicine, cancer/testis antigens, human leukocyte antigen, MHC, major histocompatibility complex, PDX, patient-derived xenograft tumors, peptidome

## Abstract

Personalized cancer immunotherapy targeting patient-specific cancer/testis antigens (CTA) and neoantigens may benefit from large-scale tumor human leukocyte antigen (HLA) peptidome (immunopeptidome) analysis, which aims to accurately identify antigens presented by tumor cells. Although significant efforts have been invested in analyzing the HLA peptidomes of fresh tumors, it is often impossible to obtain sufficient volumes of tumor tissues for comprehensive HLA peptidome characterization. This work attempted to overcome some of these obstacles by using patient-derived xenograft tumors (PDX) in mice as the tissue sources for HLA peptidome analysis. PDX tumors provide a proxy for the expansion of the patient tumor by re-grafting them through several passages to immune-compromised mice. The HLA peptidomes of human biopsies were compared with those derived from PDX tumors. Larger HLA peptidomes were obtained from the significantly larger PDX tumors as compared with the patient biopsies. The HLA peptidomes of different PDX tumors derived from the same source tumor biopsy were very reproducible, even following subsequent passages to new naïve mice. Many CTA-derived HLA peptides were discovered, as well as several potential neoantigens/variant sequences. Taken together, the use of PDX tumors for HLA peptidome analysis serves as a highly expandable and stable source of reproducible and authentic peptidomes, opening up new opportunities for defining large HLA peptidomes when only small tumor biopsies are available. This approach provides a large source for tumor antigens identification, potentially useful for personalized immunotherapy.

## Teaser

Sufficient tumor tissues are often not available for large HLA peptidome analysis. We demonstrate here that using patient derived xenograft (PDX) tumors are a useful source of large tumors for obtaining detailed and authentic HLA peptidomes that enable identification of many tumor antigens and even neoantigens of potential usefulness for personalized cancer immunotherapy.

## Graphical Abstract

**Figure:**
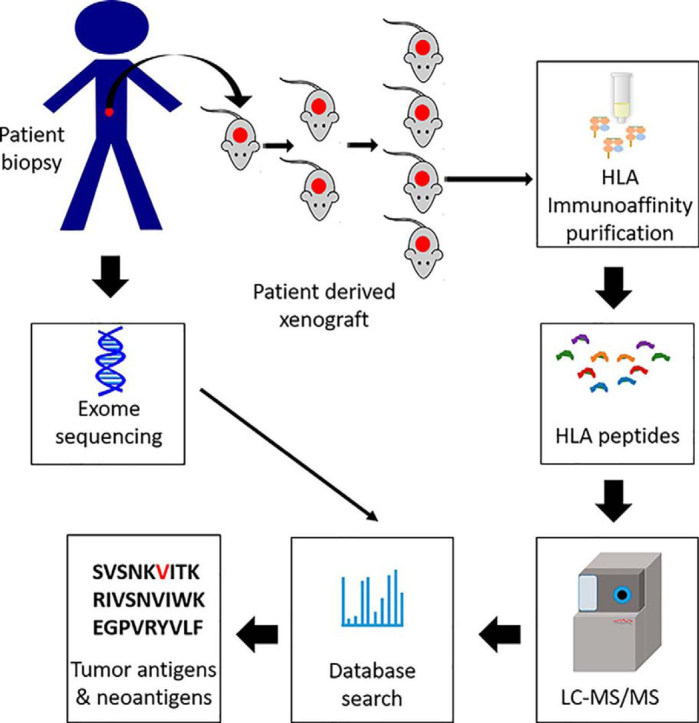

**Highlights**

• Sufficient tumor tissues are often unavailable large HLA peptidome discovery.
• Using patient derived xenograft (PDX) tumors can overcome this limitation.
• The large PDX HLA peptidomes expand significantly those of the original biopsies.
• The HLA peptidomes of the PDX tumors included many tumor antigens.

The widespread loss of gene expression control and the abundance of mutations within cancer cells leads to expression of abnormal cancer-specific proteins, against which personalized anti-cancer immunotherapies can be formulated. Once identified, the cancer-specific antigens can be administered as synthetic peptides, RNA, or DNA, in conjunction with appropriate adjuvants (1, 2). Such therapies are capable of breaking tumor-induced immune tolerance, and potential rejection of the tumors and cure (3). Indeed, mounting evidence has been showing the promising therapeutic impact of immunotherapies, mostly based on immune checkpoint inhibitors, in management of solid tumors, such as melanoma and lung (4, 5, 6, 7, 8). Other immunotherapy modalities are based on adoptive cell transfer (9), CAR-T (10), and active vaccination with tumor-associated antigens (TAA), such as cancer-testis antigens (CTA) (11, 12) and neoantigens (1, 3, 13, 14, 15, 16, 17). Neoantigens administered as long peptides or RNA molecules to advanced-stage melanoma patients, were associated with excellent cure rates, superior even to those obtained by immune checkpoint inhibitors alone (14, 17, 18).

To mount an effective anti-cancer immune response, patient T cells must recognize and react to tumor antigens presented on the tumor cells in the context of major histocompatibility complex molecules (MHC, or in humans, human leukocyte antigens, HLA). HLA class I presents peptides derived from proteins degraded within the cell and is expressed by most nucleated cells in the body. The HLAs are the most polymorphic proteins in the human population, and each HLA allomorph presents a unique set of peptides, called the HLA peptidome, immunopeptidome, or HLA ligandome. These HLA peptidomes are composed of tens of thousands of peptides, whose repertoires are mostly shaped by the schemes of protein synthesis and degradation of the cells (19, 20, 21).

The potential expression of cancer-specific antigens (CTAs and neoantigens) is generally identified by combining exome and transcriptome analyses. The generated data are used for *in-silico* prediction of HLA peptide sequences, relying on the known consensus sequence motifs of the patients' HLA allotypes. The predicted peptides are prioritized according to their gene expression levels and then tested for their immunogenicity with patient-derived antigen presenting cells and T cells (17, 22). Yet, this approach is not adequately efficient in identifying the peptides that are presented by the HLA molecules or their immunogenicity levels (23, 24, 25, 26). Therefore, direct biochemical identification of the peptides presented by the HLA molecules of patient tumor cells may help prioritize the candidate peptides for testing as potential immunotherapeutics (26, 27, 28, 29, 30), reviewed in (25, 31).

To facilitate the discovery of CTA and neoantigens HLA peptides, advanced HLA peptidome methodologies, based on immunoaffinity purification of the HLA molecules followed by capillary chromatography combined with online tandem mass spectrometry (LC-MS/MS) (32, 33) can be applied to establish patient tumor-specific large HLA peptidome data sets (25, 26, 28, 29, 34, 35). Between 5000 and 10,000 different HLA peptides, can be obtained from about 10^8^ cells, and are needed to identify just a few dozen CTA-derived peptides and one or two neoantigens. This is because of the scarcity of mutations in the protein coding sequences, and the low probability that any of them will be presented on the HLA molecules (13). In most cases, the available amounts of tumor biopsy tissues do not contain enough cells (36, 37, 38) as needed for such large HLA peptidome analyses.

The use of patient-derived xenografts (PDX) may overcome the limited availability of tumor-biopsy material for HLA peptidome analysis because they allow for practically unlimited expansion of tumor volume. These tumors, commonly used as *in vivo* models for testing drug responses and cancer therapeutics, are propagated in immune-compromised mice, where they better maintain the original patient tumor's gene expression patterns than in tissue culture (39, 40, 41). Additionally, PDX tumors better preserve sub-clonal tumor heterogeneity (42, 43, 44) when compared with cell lines and primary cell cultures, in which only subsets of the tumor cell populations survive and grow (45). To establish patient-derived tumors in mice, tumor sections, biopsy fragments or circulating tumor cells are collected from the patients and implanted subcutaneously or orthotopically into immune-deficient mice (46, 47, 48, 49). After reaching a specified size, the PDX tumors are surgically removed, sectioned, and re-grafted to new naïve mice for further expansion.

Here, to facilitate identification of tumor antigens from limiting amounts of patient's tumors, we harnessed PDX tumors as an expandable source of tissues for large HLA peptidome analysis. The large HLA peptidomes obtained from the PDX tumors represented the HLA peptidomes of original biopsies and enabled discovery of both CTAs and potential neoantigens. These findings highlight the potential benefits of implementing PDX mouse models into the development pipeline of personalized cancer immunotherapy.

## EXPERIMENTAL PROCEDURES

##### Experimental Design and Statistical Rationale

The HLA peptidomes of 8 different human biopsies and 19 of their derived PDX tumors were purified by immunoaffinity and analyzed by LC-MS/MS. The biopsies of patients P1 and P8 were sufficiently large and thus were analyzed in two biological assays. The PDX tumors of some patients could be obtained in sufficient sizes and sample numbers, allowing for separate biological analyses (Table I). The mass spectrometry data were analyzed by MaxQuant (50) using the ‘match between runs’ function only for the same patient samples' in order to maximize the peptide detection while avoiding co-assignment of unrelated peptides peaks. This was followed by statistical analysis using Perseus (51) were the similarities between the samples were evaluated using Pearson correlation. The heavy chains of the HLA of each immunoaffinity-purified sample were also recovered and analyzed by trypsin digest, followed by LC-MS/MS of the resulting peptides. In order to evaluate the fraction of mouse and human cells present in the PDX tumors, in-solution trypsin digest and LC-MS/MS analysis were performed on tumor extracts taken from the immunoaffinity column's flow-through. The database search was performed with both the human and mouse protein databases supplemented with a new database that contains all the abnormal sequences detected by the exome analyses performed on the human tumor biopsies, which differ from the standard human protein UniProt data bank.

**Table I:** Samples used for peptidome analysis and identified peptide number

Patient diagnosis *n* = 8		Gender	Sample and passage *n* = 28	ID	Samples weight (mg)	Total peptides identified (5% FDR)		After reverse, contaminant, length & mouse filtering		Peptide number fold change PDX vs human biopsy
						In specific sample	In specific patient	In specific sample	In specific patient	
Head and neck	Adnexal adenocarcinoma	M	Human biopsy	P1-human biopsy-1	<13*	392	734	212	488	10
			Human biopsy	P1-human biopsy-2	<13*	442		359		
			PDX p3	P1-PDX-1	260	4804	5864	4196	4949	
			PDX p3	P1-PDX-2	290	4713		4124		
			PDX p3	P1-PDX-3	135	3089		2745		
Bile duct	Cholangiocarcinoma	M	Human biopsy	P2-human biopsy	60	157		17		18
			PDX p0	P2-PDX-p0–1	355	417	536	218	309	
			PDX p0	P2-PDX-p0–2	258					
			PDX p3	P2-PDX-p3–1	287	473		280		
			PDX p3	P2-PDX-p3–2	337					
Lung	N/A	M	Human biopsy	P3-human biopsy	120	549		258		2
			PDX p0	P3-PDX	250	775		416		
Gastric	Carcinoma	M	Human biopsy	P4-human biopsy	100	1095		515		1
			PDX p1	P4-PDX	200	872		556		
Vascular	Hemangioendothelioma	F	Human biopsy	P5-human biopsy	<13*	804		643		0.1
			PDX p0	P5-PDX	100	244		80		
Head and neck	Squamous cell carcinoma	M	Human biopsy	P6-human biopsy	<13*	1146		1018		2
			PDX p0	P6-PDX-p0–1	100	1907	2660	1498	2095	
			PDX p0	P6-PDX-p0–2	1121					
			PDX p0	P6-PDX-p0–3	1163					
			PDX p3	P6-PDX-p3–1	1086	2068		1671		
			PDX p3	P6-PDX-p3–2	1289					
Bladder	Sarcomatoid carcinoma	M	Human biopsy	P7-human biopsy	<13*	437		344		2
			PDX p0	P7-PDX	120	784		715		
Pancreatic	Adenocarcinoma	F	Human biopsy	P8-human biopsy-1	50	131	1241	86	985	2
			Human biopsy	P8-human biopsy-2	1177	1182		951		
			PDX p1	P8-PDX-p1	1079	1387	2635	1129	2122	
			PDX p2	P8-PDX-p2	120	1988		1765		
			PDX p3	P8-PDX-p3	1142	1503		1189		


* Needle biopsy-exact weight unknown. Indicated weight is for a 14G needle which results in <13 mg cores.

##### Tumor Sample Collection and Handling

Tumor samples were obtained from the bio-bank in the Azrieli Faculty of Medicine, Bar Ilan University, Safed, Israel. The samples were collected from patients with informed consent, according to the approval and regulations of the Bar Ilan University ethics committee. Medical history and disease progression were recorded for each patient and anonymized. Tumor biopsies or resection specimens were placed in cold DMEM medium with 10% fetal bovine serum (FBS) and 1:100 penicillin/streptomycin antibiotics, maintained on ice during transportation to the laboratory and maintained at 4 °C until processing. Sections of tumor material were grafted to immunodeficient mice, whereas the remaining sections were flash-frozen in liquid nitrogen or preserved in 90% FBS with 10% DMSO and gradually frozen to −80 °C in isopropanol-containing freezing containers and stored in liquid nitrogen.

##### PDX Model

All procedures were conducted in accordance with the National Institutes of Health Guide for Care and Use of Laboratory Animals and approved by the Animal Ethics Committee of Bar-Ilan University. Tumor sections were washed in sterile saline and cut to 1 mm^3^ pieces, or mechanically dissociated (macerated) by GentleMACS (Miltenyi Biotech, Bergisch Gladbach, Germany), and grafted orthotopically to anesthetized 6–9-week-old, gender-matched immunodeficient mice (NSG = NOD.Cg-Prkdc^scid^Il2rg^tm1Wjl^/SzJl or NRG = NOD.Cg-Rag1^tm1Mom^Il2rg^tm1Wjl^/SzJ). Mice were weighed and evaluated twice a week for their general condition, tumors were detected by palpation, and measured by caliper. Mice fulfilling end point criteria (defined as weight loss >20% or tumor volume >1500 mm^3^) were euthanized by cervical dislocation or decapitation and the tumor was harvested under aseptic conditions. Excess tissues were then removed, and the tumor was sectioned by scalpel. Tumor sections were cryopreserved in 10% DMSO in FBS solution and stored in cryopreservation CryoTube vials (Thermo Fisher Scientific, Waltham, MA) placed in Mr. Frosty freezing containers (Thermo Fisher Scientific) to allow for a gradual temperature decline to −80 °C (viable preservation). Other sections were flash-frozen in liquid nitrogen (for molecular analysis). Fresh sections of the tumors were mechanically dissociated by GentleMACS and re-grafted to new mice by subcutaneous injection.

##### DNA Purification and Quantification

Paraffin blocks (FFPE) were prepared from tumor sections preserved in 4% PFA and slides were stained with Hematoxylin and Eosin (H&E) solution or with anti-HLA class I antibodies (H-300) (sc-25619; Santa Cruz Biotechnology, Dallas, TX). The stained slides were evaluated by an independent pathologist and compared with the known diagnosis of the patient's cancer. Tumor morphology and tumor cell clusters were marked by the pathologist, to enable enriched tumor DNA purification. DNA was extracted from the FFPE slides by scraping the marked human tumor cells clusters into microfuge tubes. Genomic DNA was extracted according to the manufacturer's instructions, using the Maxwell 16 FFPE Plus LEV DNA Purification Kit (Promega, Madison, WI) and the Maxwell MDxrobot (Promega). Qubit analysis was performed on each sample using Qubit dsDNA HS Assay Kit (Invitrogen, Carlsbad, CA) and the Qubit 3.0 Fluorimeter (Invitrogen).

##### Exome Sequencing and Variant Sequences Detection

DNA isolated from tumor samples was sheared to 150–200bp fragments by sonication in the Covaris Adaptive Focused Acoustics system (Covaris, Matthews, NC), according to the manufacturer's instructions. Sizing, quantitation, and quality control of the fragmented DNA were assessed by the Agilent 2100 Bioanalyzer system (Agilent Technologies, Santa Clara, CA). To construct indexed libraries, the NEBnext ultra kit (New England Biolabs, Ipswich, MA) was used to attach the Illumina sequencing adaptors (Illumina, San Diego, CA). To capture the genome interval with a target size of 50 Mb, a SureSelect v5 targeted capture library (Agilent Technologies) encompassing all exons of 21,522 genes, was used. The products were sequenced on an Illumina HiSeq 2500 instrument (Illumina) with 2 × 150 paired-end reads.

The identification of abnormal sequences was performed by GeneSort (Hertzlya, Israel) using their proprietary pipeline. Because healthy tissues of the patients were not available for exome analysis, HuVarBase iitm.ac.in/bioinfo/huvarbase/index.php, (52) was used to distinguish between potential neoantigens and SNPs.

##### HLA Peptide Purification

After tumor maceration on ice, the extracts were mixed with lysis buffer comprised of PBS supplemented with 0.25% sodium deoxycholate, 0.25 mm iodoacetamide, 1 mm EDTA, 1:200 protease inhibitors mixture (Sigma, St. Louis, MO), 1 mm PMSF, and 1% octyl-β-d-glucopyranoside. The tissue lysates were shaken gently for one hour at 4 °C, on a shaking table, and subsequently cleared by centrifugation at 4 °C and 47,580 × *g*, for 60 min (Sorval RC 6+ centrifuge, Thermo Fisher Scientific). HLA-I molecules were immunoaffinity-purified from the cleared lysate with the pan-anti-HLA class I monoclonal antibody W6/32, covalently bound to Protein A Sepharose resin with dimethylpimelimidate (53). The affinity columns were preconditioned with 2 column volumes of 0.1 N acetic acid, and next with 20 mm Tris-HCl, pH 8. After passing the extracts by gravity flow, the columns were washed with 400 mm NaCl, 20 mm Tris-HCl pH 8, followed by another wash with 20 mm Tris-HCl, pH 8. HLA class I molecules with their bound peptides were eluted with two column volumes of 1% TFA.

The recovered peptides were desalted, concentrated, and separated from the HLA molecules by reversed-phase fractionation, using disposable MicroTip Columns C-18 (Harvard Apparatus, Holliston, MA). The peptides were then eluted with 30% acetonitrile in 0.1% TFA, whereas the HLA heavy chains and other interacting proteins were recovered with 80% acetonitrile in 0.1% TFA, as previously described (54). The peptides were dried by vacuum centrifugation and then dissolved in 0.1% TFA for analysis by capillary chromatography combined with tandem mass spectrometry (LC-MS/MS). The protein fractions (80% acetonitrile fraction) were dried in a similar fashion and trypsinized for LC-MS/MS analysis. The immunoaffinity column's flow-through containing all of the tissues' remaining proteins, was collected and trypsinized as well for LC-MS/MS analysis, in order to estimate the ratio of murine to human cells in the PDX tumors.

##### Mass Spectrometry

Recovered HLA I peptides were resolved by capillary chromatography using an UltiMate 3000 RSLC or an Easy nano LC-1000 nano-capillary UHPLC, coupled by electrospray interface, to a Q-Exactive-Plus mass spectrometer (Thermo Fisher Scientific). The HLA peptides were eluted with a linear, two-hour, 5–28% acetonitrile gradient in 0.1% formic acid, at a flow rate of 0.15 μl/min. The 10 most intense ions in each full-MS spectrum, with single to triple-charged states, were selected for fragmentation by higher energy collision dissociation (HCD), at relative energy of 25. Ion times were set to 100 msec, automatic gain control (AGC) target was set to 3×10^6^ for the full MS, and to 1×10^5^ for ms2. The intensity threshold was set at 1×10^4^. For the tryptic peptides, the settings were the same as for the HLA I peptides, but double to seven charged states were selected for fragmentation. The tryptic peptides from the 80% acetonitrile fraction described above were analyzed in the LTQ OrbitrapXL mass spectrometer (Thermo Fisher Scientific) fitted with a capillary HPLC (Eksigent, Dublin, CA). The tryptic peptides were eluted using a linear gradient of 7–40% acetonitrile in 0.1% formic acid. The flow rate of the gradient program was 0.25 μl/min, for 2 h. The 7 most intense ions were fragmented by collision-induced disassociation (CID), at a relative collision energy of 35. The MS ion time was set to 100 msec, AGC target to 5×10^5^, and to 3×10^4^ for MS2. The tryptic peptides of the tumor extracts recovered from the immunoaffinity column's flow-through were analyzed in a Q-Exactive HFX mass spectrometer (Thermo Fisher Scientific) fitted with capillary chromatography using an UltiMate 3000 RSLC. The tryptic peptides were eluted using a linear 3 h gradient of 5–28% acetonitrile in 0.1% formic acid at a flow rate of 0.15 μl/min. The 30 most intense ions were fragmented by high collision dissociation (HCD), at a relative collision energy of 27. The MS/MS ion time was set to 30 msec, AGC target to 1×10^5^ for MS2, and 3×10^6^ for MS1.

##### Data analysis

Peptides were identified and quantified using the MaxQuant 1.5.8.3 and 1.6.0.16 (50) software tool with the Andromeda search engine (55). Peptide identifications were performed with the human section of the UniProt database, updated on April 2017, which contains 70,946 proteins (70,965 entries), the mouse section, updated on December 2016, which contains 50,306 proteins (50,331 entries) (www.uniprot.org), the mutation/variants database constructed from each patient's exome analysis (2602 entries) and the contaminant database of the MaxQuant software. The settings for the Andromeda search engine (55) were mass tolerance 4.5 ppm for the precursor masses, and 20 ppm for the fragments, “No-enzyme” and 5% false discovery rate (FDR); N-terminal acetylation, methionine sulfoxidation, carbamidomethyl and cysteinyl were set as variable modifications. The database search was based on identification with 5% FDR, to increase the numbers of identified HLA ligands (by about 2-fold) rather than the use of 1% FDR, assuming that selected peptides' sequences should be further validated for clinical use. For the tryptic peptides recovered from the 80% acetonitrile fraction of the proteome of the tumor extracts, the settings were “specific” for trypsin digest with two missed cleavages and 1% FDR; N-terminal acetylation and methionine sulfoxidation for the variable modifications and cysteine carbamidomethyl for fixed modification. Only unique peptides for protein quantification was set as an additional setting for the tumor extracts. Identifications were performed using the human and mouse section, as described for the HLA peptides. To improve identifications for the HLA peptides and the 80% acetonitrile fraction, the MaxQuant analyses of each patient's human biopsy and PDX tumor samples were analyzed separately, with a “match between run” option selected. To improve identification rates of the 80% acetonitrile fraction tryptic peptides, the protein sequences of the HLA allomorph that were not expressed by the patients were removed from the FASTA databases, and only the patient-specific HLA amino acid sequences (and mouse MHC *H2*) were inserted as a separate FASTA file. The HLA and *H2* sequences were obtained from the IMGT database (ebi.ac.uk/ipd/imgt/hla/) (56) and from UniProt, respectively. Statistical analysis and graphical display of the results were performed with the Perseus software tool (version 1.5.6.0) (51). Peptides identified as reverse decoy peptides, common contaminants, shorter than 8 amino acids, longer than 14 and peptides identified as mouse only, were excluded. The GibbsCluster-2.0 Server tool (cbs.dtu.dk/services/GibbsCluster/) was used to cluster the HLA peptide sequences of each patient into sequence motifs, in the patient-derived and PDX tumors, separately. NetMHCpan server (57, 58, 59) was used to define the predicted binding affinities of the peptides and to associate them with their presenting HLA allomorphs.

##### Selection of Tumor Antigens

The list of putative CTAs constructed by Shraibman *et al.* (34, 60) was based on the cancer tumor database (CT gene database, cta.lncc.br) (61) (data accumulated between the years 2005–2009) and TANTIGENE (Tumor T cell Antigen Database) (cvc.dfci.harvard.edu/tadb) (62). The list was further refined using the gene expression patterns of “BioGPS” database. The genes defined as CTAs are generally expressed in germline, embryonic and placenta cells only, and are expressed at levels below a threshold of nine gcrma units (expression units using background adjustment: GC content adjusted with Robust Multiarray Average, as described in the BioGPS website) in all normal and essential tissues. The genes were given expression scores according to the gcrma units: score 1: not expressed in any healthy (not-testis) tissues; 2: minor expression in a few normal tissues; and 3: wide-spread low expression in different healthy tissues, score 9 indicates insufficient data available on the specific gene, and score 10 represents higher expression in some normal tissues (60). Subsequently, prioritization of the CTAs was done using the Human Protein Atlas (63)(proteinatlas.org). Each CTA was ranked according to the level of its RNA expression in healthy tissues, compared with the immune-privileged sites (testes). The rationale for this prioritization was that CTAs should not have higher expression levels in normal tissues than NY-ESO-1 (64, 65) because this CTA was shown to have clinical benefits without inducing major adverse effects (65, 66, 67). CTAs with RNA expression only in immune-privileged sites (*i.e.* testis, endometrium, placenta) and in no other tissues (similar to NY-ESO-1) were marked in Table III as excellent candidates (+++); CTAs with expression in immune-privileged sites, with low levels of expression in other tissues (big difference between the levels), were marked as very good (++); CTAs with expression in immune-privileged sites with small differences in their levels of expression relative to other tissues were marked as good (+); highest RNA expressions in tissues that are not immune-privileged were marked as sub-optimal (−).

**Table III:** Peptides derived from CTA proteins detected in the tumors' HLA peptidomes

Tumor antigens	Protein name	Patient diagnosis	Sample ID	HLA peptide	HLA allomorph	NetMHCpan rank prediction	Bio-GPS score	HPA based prioritization
AKAP13	A-kinase anchor protein 13	Head and neck Adnexal adenocarcinoma	P1-PDX	ETFGGFDSHQM	A*26:01	0.1576	2	+
		Pancreatic adenocarcinoma	P8-PDX + Human biopsy					
FMNL1	Formin-like protein 1	Head and neck Adnexal adenocarcinoma	P1-PDX	NHIGWVQEF	C*06:02	0.7677	2	−
			P1-Human biopsy	VPPPPPPPP	non	6.7224		
		Vascular Hemangioendothelioma	P5-Human biopsy	HPACVNEIAL	B*35:02	0.0332		
MDM2	E3 ubiquitin-protein ligase Mdm2	Pancreatic adenocarcinoma	P8-PDX	YTMKEVLFY	A*29:02	0.0061	3	−
		Head and neck Adnexal adenocarcinoma	P1-PDX		A*01:01	0.0169		
				IPASEQETL	B*35:02	0.0048		
		Head and neck Squamous cell carcinoma	P6-PDX	DEKQQHIVY	B*18:01	0.0236		
				DEVYQVTVY		0.0097		
			P6-PDX + Human biopsy	SEQETLVRP	B*41:01	0.7923		
MET	Hepatocyte growth factor receptor	Head and neck Squamous cell carcinoma	P6-PDX + Human biopsy	SYIDVLPEF	A*23:01	0.0158	1	+
		Sarcomatiod carcinoma in bladder	P7-PDX	EVLLTSISTF	A*25:01	0.1139		
ODF2	Outer dense fiber protein 2	Head and neck Adnexal adenocarcinoma	P1-PDX	QAHLEVQQL	Non	2.5962	3	++
		Lung	P3-PDX	KILDLETQL	A*02:01	1.3753		
TTK	Dual specificity protein kinase TTK	Head and neck Adnexal adenocarcinoma	P1-PDX	IIDPNHEIEF	A*01:01	1.6605	2	++
FTHL17	Ferritin heavy polypeptide-like 17	Pancreatic adenocarcinoma	P8-PDX	VNQSLLDLY	A*29:02	0.9931	1	++
HSPA1A	Heat shock 70 kDa protein 1A	Gastric Carcinoma	P4-PDX	TIDDGIFEV	A*02:01	0.083	2	−
BIRC 5	Baculoviral IAP repeat containing 5	Head and neck Adnexal adenocarcinoma	P1-PDX	CPTENEPDL	B*35:02	0.2024	2	+
PRAME	Preferentially expressed antigen in melanoma	Pancreatic adenocarcinoma	P8 PDX	SHCSQLTTLSF	B*38:01	0.4077	2	++

##### HLA Typing

DNA samples from the PDX tissues were typed by the Tissue Typing and Immunogenetics Laboratory, Hadassah Medical Organization for three loci: HLA A, B, and C, in intermediate resolution by the PCR- Sequence Specific Oligonucleotide Probe (SSOP) method of Immucor life codes (Peachtree Corners, GA) according to manufacturer instructions.

## RESULTS

**Fig. 1:**
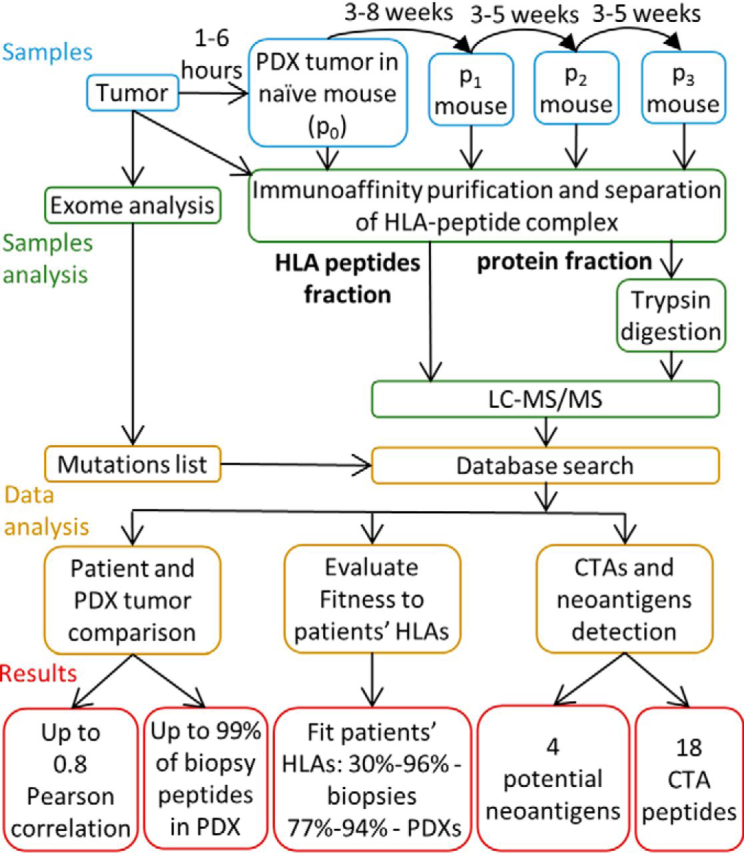
**Experimental procedure and study design.** Sample types and data analysis scheme of HLA peptidomes of PDX and human biopsies.

**Fig. 2:**
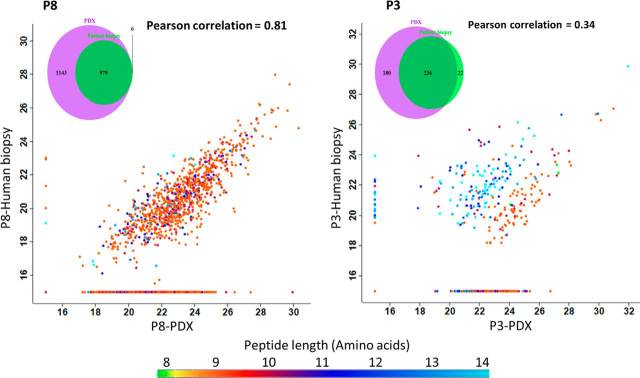
**Similarity between HLA peptidomes of patient biopsies and PDX tumors.** Scatter plots showing the level of similarity between the HLA peptidomes of the patients' biopsies and the PDX tumors. The dots represent the relative LC-MS signal intensities for each peptide on a log2 scale, with peptide length indicated in color, as per the scale at the bottom. The group of HLA peptides detected in only one of the samples is indicated on the vertical/horizontal lines assigned with the imputed arbitrary numbers of 15. The Venn diagrams demonstrate the increase in the number of HLA peptides detected in the human biopsies (green) relative to the PDX tumor (purple) (after filtering for contaminants, length, and human sequences). The samples displayed in these scatter plots are of patient P8 (pancreatic adenocarcinoma) combined human biopsies 50/1177 mg, *versus* combined PDX tumors 1079/120/1142 mg and patient P3 (lung) biopsy (120 mg) *versus* PDX tumor (250 mg).

The HLA peptidome analyses were performed in parallel, using macerated and detergent-solubilized human biopsies and PDX tumor tissues. The HLA molecules were immunoaffinity purified and the bound peptides were analyzed by LC-MS/MS (Fig. 1). The main advantage of PDX tumors as a source for HLA peptidome seems to emanate from the availability of larger tissue samples relative to patients' tumor biopsy specimens, thus enabling identification of an average of 5-fold more peptides from most PDX tumors (Tables I and supplemental Table S2, Figs. 2 and supplemental Fig. S1).

Importantly, the HLA peptidomes recovered from the PDX tumors largely resembled the peptidomes of the human biopsies, both in their repertoires of peptides and in the relative LC-MS-determined intensities of the individual peptides (Figs. 2 and supplemental Fig. S1, and supplemental Table S2). Pearson correlations between the human biopsy and their derived PDXs reached 0.83, with better correlations and more authentic HLA ligands identified from the larger human biopsies and PDX tumor tissue samples. Furthermore, up to 99% of the HLA peptides detected in the human biopsies were also identified in their respective PDX tumors (Figs. 2 and supplemental Fig. S1).

**Fig. 3:**
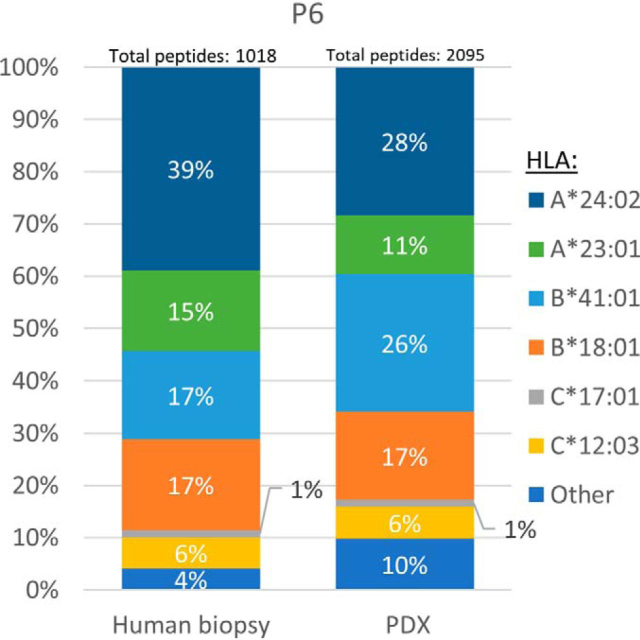
**An example of the distribution of peptides according to their HLA allotypes in biopsy and PDX tumors.** Shown are the percentages of HLA peptides that fit the sequence motifs of the HLA allotypes of patient P6 according to NetMHCpan.

Peptides are assumed to be true HLA ligands when their sequences fit the consensus sequence motifs of their presenting HLA allomorphs. NetMHCpan server was used to suggest distinctions between true HLA ligands and non-ligands, *i.e.* contaminating peptides that do not fit any of the patients' known HLA allotypes supplemental Table S1 and S2). Between 18 and 96% of the human biopsies and between 54 and 92% (excluding patient P5) of the PDX tumor HLA peptides matched the sequence motifs of the patients' HLA allotypes. Furthermore, the ratios of these peptides were similar to those observed in the human biopsies (Figs. 3 and supplemental Fig. S2). Unfortunately, not all of the consensus peptide sequence motifs of these patients' HLA allomorphs are known (especially of the HLA-C allotypes), thus leaving a wide range of peptides unassigned to specific patients' HLAs (4–82% in the human biopsies and 8–46% in the PDX tumors (Figs. 3 and supplemental Fig. S2).

The peptidomes of the human biopsies and PDX tumors were separated into subgroups by Gibbs clustering to evaluate their fitness to their presenting HLA allomorphs (68), followed by sequence cluster fitting to the sequence motifs of the HLA allotypes (69). The analysis was performed with 9 amino acid-long peptides, and the number of clusters was set according to the number of allotypes detected by the HLA typing analysis. For example, the sequence clusters fit the consensus sequence motifs of five of the allotypes of patient P6 (HLA-A*24:02, A*23:01, B*41:01, B*18:01, C*12:03, supplemental Table S1); none of the clusters fit its C*17:01 (supplemental Fig. S3). Yet, in some of the biopsies, the Gibbs clustering was less effective. For example, in Patient P4 samples, in which most of the biopsy peptide sequences did not cluster well, the peptidome of its derived PDX tumor clustered significantly better (supplemental Fig. S4). These observations further support the suggestion that PDX tumors provide a useful and reliable source for HLA peptidome analysis, which is often better if only limiting amounts of tumor biopsies are available. An exception to this trend was the hepatic hemangioendothelioma tumor (P5). For a yet unknown reason, the HLA peptidome analysis of the biopsy resulted in the identification of 643 peptides as compared with the PDX tumor, which gave rise to only 80 peptides (Table I). Accordingly, larger amounts of HLA molecules were recovered from patient P5 biopsy, relative to its PDX tumor (supplemental Fig. S5).

**Fig. 4:**
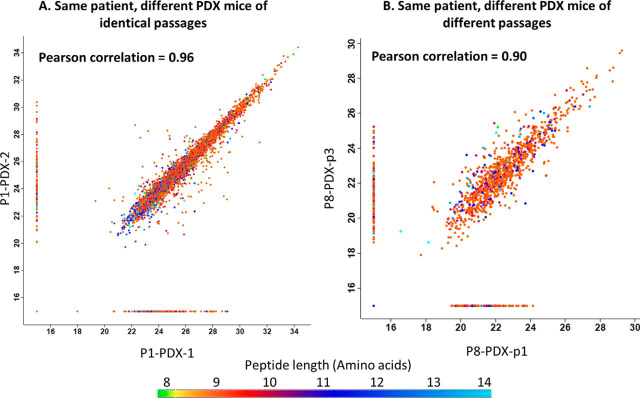
**Similar HLA peptidomes were detected in different PDX tumors originating from the same patient.** Each dot represents the relative LC-MS signal intensity of a single peptide, on a log2 scale, with the color indicating the peptide length, as per the scale at the bottom. The group of HLA peptides detected in only one of the samples is indicated on the vertical/horizontal lines with the imputed arbitrary numbers of 15. *A*, PDX tumors from the third passage of patient P1 (head and neck adnexal adenocarcinoma); *B*, PDX tumors from the first passage *versus* third passage of patient P8 (pancreatic adenocarcinoma).

**Fig. 5:**
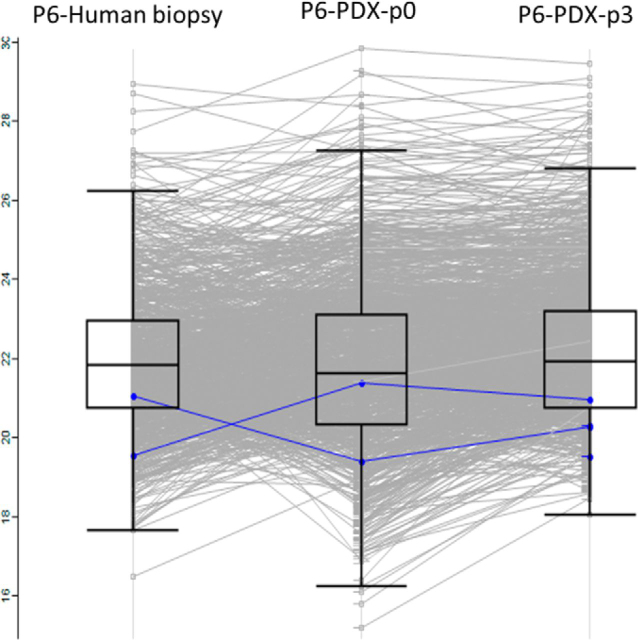
**HLA peptidomes from different passages are like the human biopsy of patient P6.** Each line represents the relative LC-MS signal intensity of a single peptide and the mean intensities are indicated by the boxes on a log2 scale, with the color indicating the CTAs detected in these samples. The CTAs marked in blue are MDM2 and MET.

The level of reproducibility of the analytical methodology was investigated using PDX tumor samples originating from the same patient tumor, grafted in parallel and in subsequent passages of mice. The HLA peptidomes recovered from different mice of the same passage were similar, with Pearson correlations reaching 0.96. Examples of such highly reproducible peptidomes were the three PDX tumors derived from patient P1 (head and neck adnexal adenocarcinoma) of the same third passage (Figs. 4*A* and supplemental Fig. S6). In Addition, the PDX tumors of different passages of patient P8 (cholangiocarcinoma) passage-one and three had a Pearson correlation of 0.90, passages two and three had 0.79, and the Pearson correlation between passage one and two was 0.78 (Figs. 4*B* and supplemental Fig. S6). This observation demonstrates the similarity of the HLA peptidomes of the different PDX passages. Furthermore it seems that there were no PDX passages that were preferably similar to the human biopsy (Fig. 5).

One of the main difficulties in HLA peptidome analysis is the prevalence of sample contamination with peptides that co-isolate with the true HLA ligands (70), as was indeed observed here in both human biopsies and PDX tumors (Table I). Such peptide contaminations are detected because of the exquisite sensitivity of the LC-MS/MS and are observed to larger extents in small tissue samples and in tissues containing low levels of HLA molecules. Such contaminations are likely caused by the mild washing conditions of the immunoaffinity columns, intended to prevent loss of the lower affinity HLA ligands. Indeed, in some small biopsy samples, most of the identified peptides were likely contaminating peptides, as determined by their lengths distribution. Most PDX tumors were larger, providing more true HLA ligands of 9 amino acids long, which fit the consensus motifs of their HLA allomorphs (supplemental Fig. S7).

PDX tumors contain both human and mouse cells (71), therefore, contamination of the HLA peptidome by some mouse peptides is unavoidable. The proteomes of the PDX tissues were analyzed from the immunoaffinity purification column flow-through to estimate the ratios of murine and human cells in the tumor. The analysis revealed that the ratio was about 1:1 in all the PDX tumors (supplemental Table S4). To distinguish between peptides originating from human *versus* mouse proteins, both the human and the mouse sections of the UniProt database were used for the Andromeda database search. For all analyses, the mouse-only sequences were removed from the lists of identified HLA peptides, whereas the shared human and mouse sequences were retained.

To further investigate the sources of contamination of PDX tumor HLA peptidomes with mouse peptides, we evaluated the relative amounts of MHC *H2* molecules (the MHC heavy chains) that co-purified with the human HLA molecules during the immunoaffinity purification. The iBAQ values (72) were defined by the MaxQuant software using the amino acid sequences of the HLA of each patient and the relevant MHC *H2* sequences of the immunodeficient SCID mouse strain (H-2K^d^ and H-2D^b^), searched in addition to the human and mouse UniProt protein database. For example, in the patient P1 biopsy, no *H2* molecules were detected, whereas the affinity-purified HLA of the PDX tumors contained small amounts of such contamination (iBAQ = 8×10^8^) relative to the PDX's HLA molecules, which showed iBAQ of 5×10^10^ (supplemental Fig. S8 and supplemental Table S3). The small amount of co-purifying MHC H2 molecules could also explain the 82 mouse peptides (out of 407 mouse peptides from all samples) that were detected among the HLA peptidomes, and fitted the MHC H2 sequence motifs of the mouse (supplemental Table S2). These relatively small numbers of mouse only peptides that were identified among the HLA peptides were distributed with different intensities, MaxQuant scores and NetMHC ranks, similarly to the Human HLA peptides (supplemental Fig. S9).

**Fig. 6:**
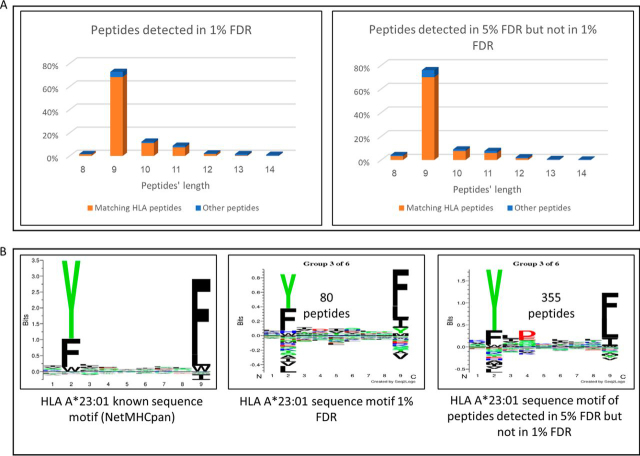
**The length distributions of the peptides and their Gibbs cluster analysis provide an indication that the newly detected peptides by use of 5% FDR analysis are mostly true HLA ligands.** The LC-MS/MS data of the HLA peptidome of patient P6 (head and neck squamous cell carcinoma) was analyzed using 1 and 5% FDR and the peptide lists identified by the 1% FDR or added by using 5% FDR were compared. *A*) Length distribution and fitness to the HLA consensus sequence motifs of the patient (colors) according to NetMHCpan. *B*) Example of Gibbs cluster representing the patient's HLA A*23:01. The number of peptides used for each cluster is indicated above the logo. (The Gibbs clusters representing the other patient's consensus sequence motifs are displayed in supplemental Fig. S10).

One of the main goals of HLA peptidome analysis is to enlarge the ‘target bank’ of tumor-specific neoantigens and CTA HLA peptides, which can be used to direct personalized antitumor immune reactions. Indeed, the combined HLA peptidome analyses of the biopsies and their derived PDX tumor tissues provided extensive lists of peptides, among which numerous CTAs and even several potential neoantigens were detected (Table II, Table III). To identify neoantigens, the protein sequences (in a FASTA format) containing the variants detected by the exome analyses of the tumors, were added to the regular UniProt databases FASTA files used for the Andromeda search. Next, HuVarBase iitm.ac.in/bioinfo/huvarbase/index.php, (52) was used to distinguish between potential neoantigens and SNPs. To enlarge the “target bank” of tumor-specific neoantigens and CTA HLA peptides, we used 5% FDR for the database search rather than 1% FDR, which is often used in HLA peptidome studies (26, 27, 28, 34, 54, 60, 73), resulting in about 2-fold enlargement of the identified HLA peptides list. Although it is clear that the use of 5% FDR enlarged also the number of false identifications, larger numbers of ‘likely HLA ligands’ were added to the list of identified peptides, as can be judged from the fitness of their sequences to the consensus sequence motifs of the patients' HLA and to the 9 amino acid preference of HLA ligands (Figs. 6 and S10). Potential neoantigens were selected using the same stringent parameters as the normal HLA peptides, including peptides with NetMHCpan scores <2. The potential neoantigen HLA peptides detected in these samples (summarized in Table II) were KYIERIITQF, derived from the Ecdysoneless cell cycle regulator, (P4-PDX), and from patient P6, PDX and human biopsy; RYFDEPVEL derived from the ADP-ribosylation factor GTPase activating protein 3, EYLTPEILEL derived from H2A clustered histone 16. The peptide FLIDKINAF (P8-PDX), derived from the MX dynamin-like GTPase 1, was found to be a likely SNP in HuVarBase and is included as an example for the filtering process. These potential neoantigens are an example of the usefulness of the PDX method for the discovery of neoantigens actually presented on the tumor cells, even with limited tumor tissue samples.

**Table II:** Mutated HLA peptides detected in tumors' peptidomes

Sample ID	Patient diagnosis	Mutated peptide	Mutation	Gene	HLA allomorph	NetMHC pan rank prediction	SNP
P4-PDX	Gastric carcinoma	KYIERIIT**Q**F	Arg → Gln	ECD	A*23:01	0.017	+^a^
P6-Human biopsy	Head and neck Squamous cell carcinoma	**R**YFDEPVEL	Ser → Arg	ARFGAP3	A*23:01	0.2796	–
P6-PDX-p3		EYLT**P**EILEL	Ala → Pro	H2AC11	A*23:01	1.1457	–
P8-PDX	Pancreatic adenocarcinoma	FLIDK**I**NAF	Val → Ile	MX1	A*26:01	0.0498	+

a SNP disease associated.

Because CTAs are more abundant, and therefore easier to detect as HLA peptides, compared with neoantigens, larger numbers of peptides derived from CTAs were identified in this analysis (Table III). In the combined analysis of all patient samples, a total of 409 candidate CTA HLA peptides, derived from 130 CTA genes (PDX and human biopsies) were identified. Of these, only 18 peptides (4.4%) were given a CTA score of 1, 2, or 3 (Table III) using the Bio-GPS database, and out of them only 7 showed a very good and good ranking using the HPA database (Experimental Procedure).

## DISCUSSION

The results presented here support the notion that PDX tumor models are a useful and adequate tissue source for large-scale HLA peptidome analysis. The tumor cells in the PDX tumor retain high HLA peptidomes similarity to those in the human biopsy. The large HLA peptidome data sets obtainable from PDX tumors may prove clinically relevant in the areas of precision medicine and patient care. However, more research is needed for clinical immunotherapy implementation of the PDX models.

In this study, we evaluated the use of PDX tumors for HLA peptidome analysis and demonstrated some of its advantages and limitations. One of the advantages of the PDX system as a model for cancer research is the provision of large amounts of tumor tissues, highly similar to the human biopsy tissue, whereas biopsy samples only provide small tissue volumes, which, resulted in identification of up to 18-fold more HLA peptides (Table I, Figs. 2 and supplemental Fig. S1). Furthermore, analysis of small-sized tissue samples also suffers from identification of a higher percentage of contaminating peptides than larger sized samples. Therefore, the PDX tumor model both enlarges the pool of target peptides and facilitates the detection of more authentic HLA ligands. This phenomenon is exemplified by the data collected from patient P6 and patient P4 (supplemental Fig. S7), whose biopsy samples carried peptides of more variable lengths as compared with the HLA peptides derived from the PDX tumor. Patient P5 was an exception, who for yet unknown reasons, had lower numbers of HLA peptides in the PDX tumors *versus* the biopsy samples. We assume that there were fewer HLA molecules in this patient's cancer cells (supplemental Fig. S5), as is known to happen often in human tumors (74). The patients' tumors contain both cancer and non-cancerous stromal and immune cells, and because it is primarily the cancerous cells that survive in the mouse environment, it is possible that tumors with low levels of HLA on the cancer cells will be manifested in the PDX tumors. We include the data on this patient's PDX to demonstrate that this model might not be suitable to all tumor types, or all patients. Further investigation of the reasons for the reduced HLA presentation in this PDX sample and what are the factors contributing to a successful PDX model is beyond the scope of this work.

A central question with regards to the clinical relevance of the PDX models is whether the tumors, from different mice, remain like the patient tumor (75). Indeed, in this research, a considerable similarity was observed between the HLA peptidomes of the different PDX tumors of each patient, which also resembled the biopsy HLA peptidomes (Figs. 2, 4*A* and supplemental Fig. S1). Furthermore, different passages remained similar to one another (Figs. 4*B* and supplemental Fig. S6), thus, HLA peptidomes of late PDX passages could be useful as well, even though it is suggested that earlier passages remain more similar to the origin (76). Fitness of the identified peptidomes to the peptide sequence motifs of the HLA allomorphs of the patients is a good indicator of the authenticity of the discovered HLA peptidomes. Indeed, the PDX tumors' HLA peptidome were very similar to patients' peptide sequence motifs (Fig. 3 and supplemental Fig. S2), and this fitness may be further refined with the discovery of more HLA-C allotypes sequence motifs. The discovery of these sequence motifs is difficult because this allomorph has low expression levels in the cells. This can be shown by the low numbers of peptides assigned to the HLA-C allomorphs (supplemental Table S2) and by the relative amounts of HLA-C molecules that were recovered in the 80% acetonitrile fraction (supplemental Fig. S7 and supplemental Table S3). The Gibbs cluster analysis demonstrated these similarities between the human biopsies and PDX tumors (supplemental Fig. S3 and S4) without the HLA allomorph reference, supporting the usefulness of this methodology. A limitation to the Gibbs cluster analysis is the input size of sequence data: the larger the number of peptides inputted, the better the cluster is. We conclude that the HLA peptidomes of the PDX tumors are relatively authentic and closely represent the landscape of HLA peptide presentation of the human biopsy (Figs. 3 and supplemental Fig. S2).

Expansion of the size of the identified HLA peptidomes using the PDX model also enhanced detection of CTAs and potential neoantigens (Table II, Table III), which could not be detected with the small human biopsy alone. This also suggests that the use of the PDX mouse model may be superior to *in vitro* primary tumor cells culturing (77), where the cells are less likely to represent the true diversity and full HLA peptide repertoire of cells existing in a patient-derived tumor. In addition, as indicated by Ben David *et al.* (75), some preexisting clones expand within the PDX tumors, because of selective pressure of the PDX microenvironment. Grafting multiple biopsies to different mice may help to uncover the diversity of antigens in sub-clones, which is potentially useful for immunotherapy. Size expansion of the identified HLA peptidomes can be also achieved by using 5% FDR (26, 27, 28, 34, 54, 60, 73) rather than the more stringent 1% FDR that is used in many modern HLA peptidome studies that employ high resolution and accuracy mass spectrometry (29, 30). The use of 5% FDR increased by about twice the HLA peptides identification (for example, additional 1343 likely HLA ligand peptides in the samples of patient P6). These additional peptides mostly conform to the 9 amino acid length rule and fitted the consensus sequence motifs of the HLA of the patient (Figs. 6 and S10). However, an increase in the number of identified peptides obtained by using 5% FDR, generated an additional 4% of false identifications (about 88 peptides out of 2212 peptides of patient P6). We, therefore, suggest that for personalized immunotherapy studies, it might be advantageous to maximize the number of possible neoantigens and CTA HLA peptides by a less stringent FDR, and delaying validation of each of the neoantigens and CTA peptides to later stages. The validation process should include the use of synthetic peptides to compare the endogenous peptide spectra found in the PDX to a synthetic peptide spectra. This way one can be certain that the detected peptides are true HLA ligands and not false positives (26, 27, 28, 29, 30, 33, 73).

The use of PDX models is known to be advantageous also in translational drug development (78), and cancer research because of the physiologically-relevant tumor microenvironment and intact endocrine systems, as well as the ability to study aspects of metastatic spread and tumor biology such as angiogenesis (61, 77, 79, 80, 81, 82). That said, PDX tumors have significant limitations that impact their translational potential. These limitations include the lack of a functional human immune system and the lack of a human microenvironment. An additional limitation that affects the usability of PDX models for personalized medicine is the long timeline to development of each model (typically 2–8 months to develop a PDX model for a preclinical study, which could be too long to be applicable for many of the patients) as do the significant expenses, and labor associated with PDX model initiation and maintenance (41, 83). Additionally, not all tumors engrafted to immunodeficient mice will have the potential to form PDX tumors and success rates for tumor engraftment vary among tumor types (84) with possible variation between labs and methods as a contributing factor (41). Although there could be technical advances to address some of these limitations (for instance, grafting the tumor tissue with the original surrounding human stroma, to maintain human aspects of the microenvironment (78), and incorporation of human immune cells in humanized models (41, 85, 86)), further studies are required to increase clinical relevance and to clarify the shortcomings of the PDX models.

The observation that the HLA peptidomes contain mouse-specific sequences can be because of nonspecific co-purification of mouse MHC molecules, cross-presentation of mouse peptides by the human cells, proteolysis of murine mAb molecules and other co-purifying proteins or false identifications by the bioinformatics software. Up to about 60% of mouse specific peptides in the PDX models fitted the patients' HLA peptide binding motif (supplemental Table S2). This could be because of overlap between the binding motifs of the patients and of the mice or may suggest that mouse peptides might have been loaded exogenously by cross presentation onto the HLA molecules of the human tumors grafted in mice (87, 88). An interesting point is the similar distribution of the NetMHCpan ranks of the mouse-only peptides compared with the NetMHCpan ranks of the human HLA peptides. In addition, the distribution of LC-MS intensities and MaxQuant scores were also like the human only peptides (supplemental Fig. S9). This suggests that some mouse peptides are possibly true ligands of the co-purifying MHC H2 molecules or are cross presented by the HLA molecules, which is less likely because of scarcity of the human antigen presenting cells in the PDX tumors. Up to 20% of the mouse peptides fitted the MHC H2 sequence with better rank in NetMHCpan than the HLA of the patients (supplemental Table S2) supporting the notion that some of these mouse peptides are ligands of MHC H2 molecules that co-purified with the HLA molecules. Such contaminations can be distinguished during subsequent sequence analysis steps by removal of mouse-only peptides from the list of identified HLA ligands, and therefore do not pose any problem for the data analysis.

Neoantigen application in personalized immunotherapy is a new and exciting clinical direction (1, 13), which has drawn much attention to HLA peptidomics as a useful tool for their detection (25, 26). The frequency of neoantigen presentation as an HLA peptide is relatively low, compared with non-mutated antigens, and therefore, their detection and identification are difficult (1, 17, 24, 26, 27, 28, 29, 69, 89, 90). The lack of healthy tissues in this study for exome analysis prevented the definition of abnormal peptides sequences as certain neoantigens derived from mutated sequences in the tumor cells and not in the healthy tissues of the same patients. Searching the SNP database helped to select those peptides that are not known SNPs and therefore can be defined as potential neoantigens. This serves as a proof of concept for the usefulness of this method. The small numbers of potential neoantigens discovered by HLA peptidome analysis stress the need to improve purification and identification efficiency, which has been significantly advanced by the PDX tumors.

The four neoantigens/variant antigens identified in this study, and the majority of CTAs' peptides fit the respective patients' HLA allomorphs peptide consensus motifs, and were of typical lengths of HLA class I peptides, *i.e.* 9 amino acids. Immunogenicity testing, requiring patient-derived T cells, is still required to validate such peptides. Regrettably, such cells were not available in this study. Significantly, CTA and potential neoantigen HLA peptide detection using PDX tumor models in mice demonstrate yet another useful implementation of PDX in the clinical development pipeline. In addition to immunotherapy, HLA peptidome from PDX models could be useful in many other fields, where the immunopeptidome could provide an insight on the immune process. PDX models are argued to be better representative of the microenvironment of the tumors, and therefore HLA peptidomes preformed from PDX models could be helpful in deepening our understanding of the changes in the HLA peptide landscape and the HLA presentation process. A good example of this could be for testing different drugs in a mice context and looking into their immunopeptidome for new emerging antigens (60).

Questions remaining unanswered pertain to the effect of the site of implantation and the tumor grafting method on the recovered PDX model HLA peptidome. Two methods were used here to generate the PDX models, *i.e.* direct grafting of tumor fragments or tumor maceration prior to injection into the mice. Although only two tumors were macerated prior to injection, they did not show distinguishable differences from the other tumors. Tumor maceration is expected to better represent the heterogeneity of the human biopsy cell populations, enabling detection of antigens present only in parts of the patient tumor. The grafting site and its microenvironment may also impact the PDX tumor and its HLA peptidome. Although the effect of the grafting site was not evaluated in this study, tumors were engrafted subcutaneously or intraperitoneally and showed no significant differences in their obtained HLA peptidomes between the two methods.

In conclusion, this preliminary study highlighted the advantages of PDX models as a source of large and authentic HLA peptidomes, containing both CTA and neo-epitopes, which may be of benefit to personalized cancer immunotherapy.

A note added in proof: after submission of this manuscript, a publication by Heather *et al.* showed the use of cell line derived Xenograft Bioreactors tumor as a tissue source for immunopeptidome analysis (91).

## DATA AVAILABILITY

The mass spectrometry proteomics data have been deposited to the ProteomeXchange Consortium via the PRIDE (92) partner repository with the data set identifier PXD016060.
