# Supplementary material for: Identification of Tumor Antigens in the HLA Peptidome of Patient-derived Xenograft Tumors in Mouse
Source: Mol Cell Proteomics. 2020 Nov 23;19(8):1360–74. doi: 10.1074/mcp.RA119.001876 (PMC8015002; doi:10.1074/mcp.RA119.001876)
Supplement: Supplementary file 1 [file mmc1.zip › 157154_2_supp_533875_qtmxd1.pdf]

Identification of tumor antigens in the HLA peptidome of patient-derived xenograft tumors in mouse

Authors: Nataly Mancette Rijensky, Netta R. Blondheim Shraga, Eilon Barnea, Nir Peled, Eli Rosenbaum, Aron Popovtzer, Solomon M. Stemmer Alejandro Livoff, Mark Shlapobersky, Neta Moskovits, Dafna Perry, Eitan Rubin, Itzhak Haviv, and Arie Admon

List of contents:

- Supplemental Figures S1-S9
- Supplemental method western blot
- Supplemental Table S1

Supplemental Figures:

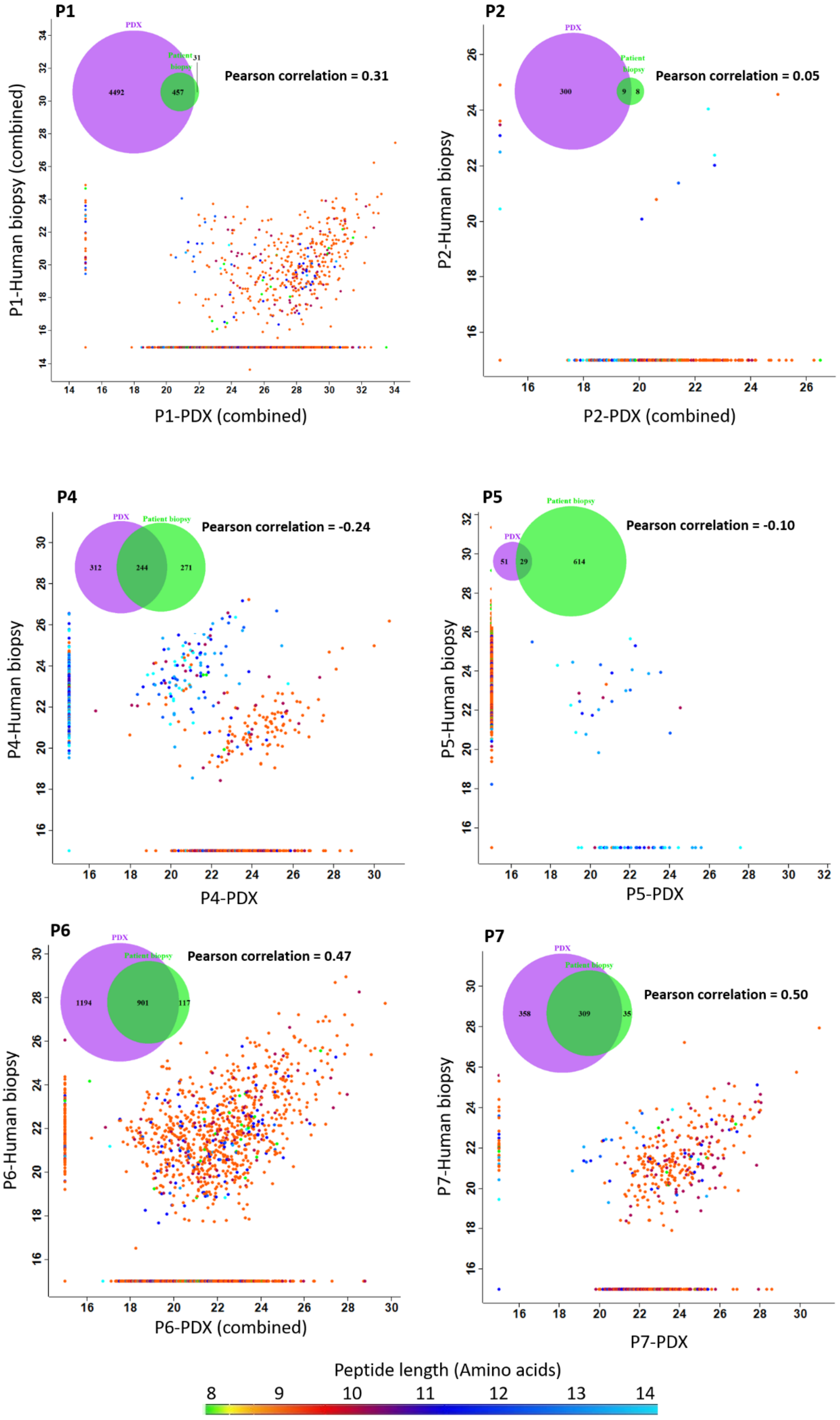

P1
PDX
Patient biopsy
4492
457
31
Pearson correlation = 0.31
P1-Human biopsy (combined)
P1-PDX (combined)
P2
PDX
Patient biopsy
300
9
8
Pearson correlation = 0.05
P2-Human biopsy
P2-PDX (combined)
P4
PDX
Patient biopsy
312
244
271
Pearson correlation = -0.24
P4-Human biopsy
P4-PDX
P5
Patient biopsy
PDX
51
29
614
Pearson correlation = -0.10
P5-Human biopsy
P5-PDX
P6
PDX
Patient biopsy
1194
901
117
Pearson correlation = 0.47
P6-Human biopsy
P6-PDX (combined)
P7
PDX
Patient biopsy
358
309
35
Pearson correlation = 0.50
P7-Human biopsy
P7-PDX
Peptide length (Amino acids)
8
9
10
11
12
13
14

Figure S1: High similarity between HLA peptidomes of patient biopsies and PDX tumors.-. Scatter plots of the LC-MS signal intensities of the HLA peptides of the tumors of patients P1, P2, P4, P5, P6 and P7. The dots represent the relative LC-MS signal intensities for each peptide detected in both samples (log2 scale), with the color indicating peptide length. The group of HLA peptides detected in only one of the two samples is indicated on the vertical/horizontal lines with imputed arbitrary numbers of 15. The Venn diagrams demonstrate the increase in the number of HLA peptides detected as a result of the use of PDX models, as well as the number of HLA peptides (after filtering) detected in both the PDX tumor and the human biopsies.

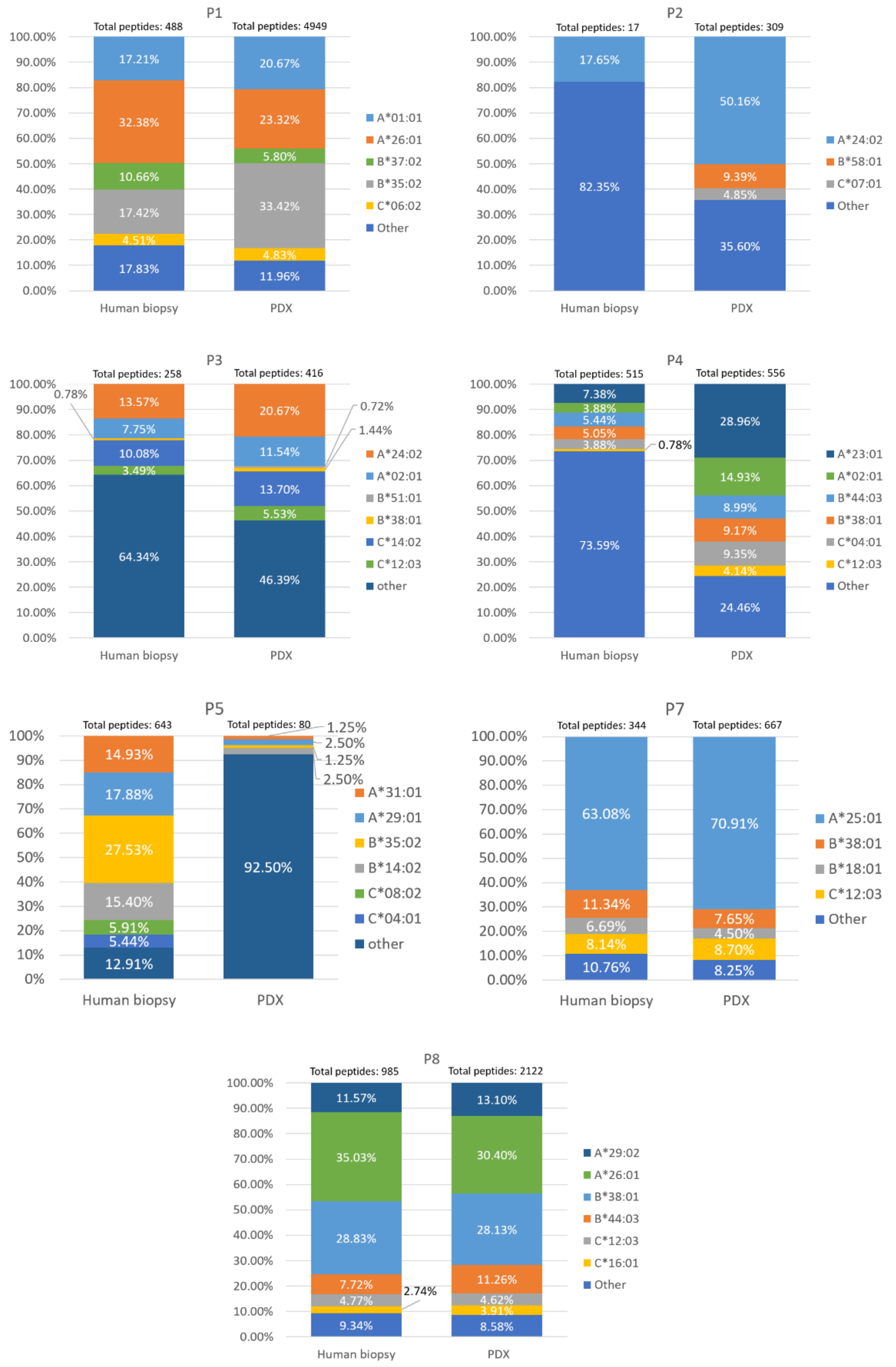

Figure S2: distribution of peptides according to their HLA allotypes in biopsy and PDX tumors: Shown are the percentages of HLA peptides that fit the sequence motifs of the HLA allotypes of patient P1, P2, P3, P4, P5, P7 and P8 according to NetMHCpan.

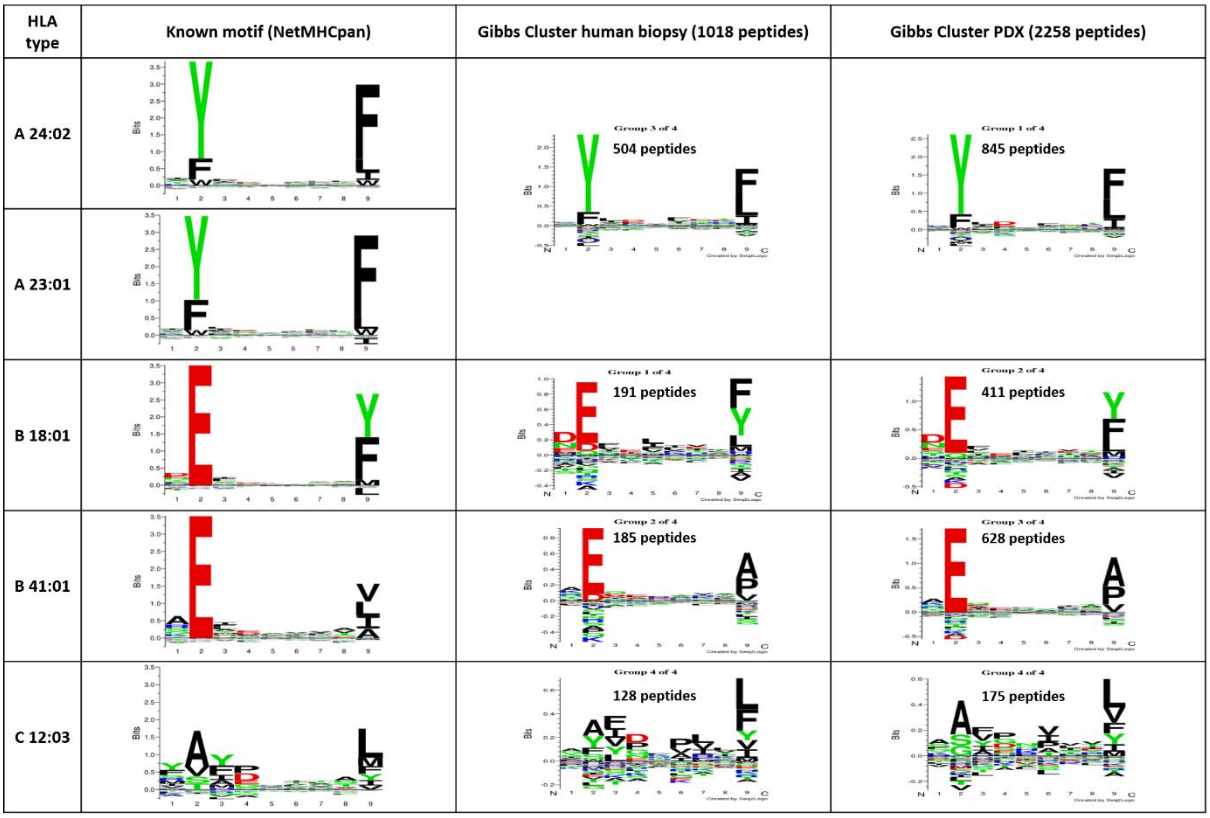

Figure S3: Gibbs cluster of the HLA peptides from patient P6 biopsy and PDX tumor in comparison to their known sequence motifs. The known consensus sequence motifs of the HLA allotypes of Patient 6 (head and neck squamous cell carcinoma) were taken from NetMHCpan and displayed next to the patient's Gibbs clustering of his biopsy and PDX tumor. The number of peptides used for each cluster is indicated above the logo.

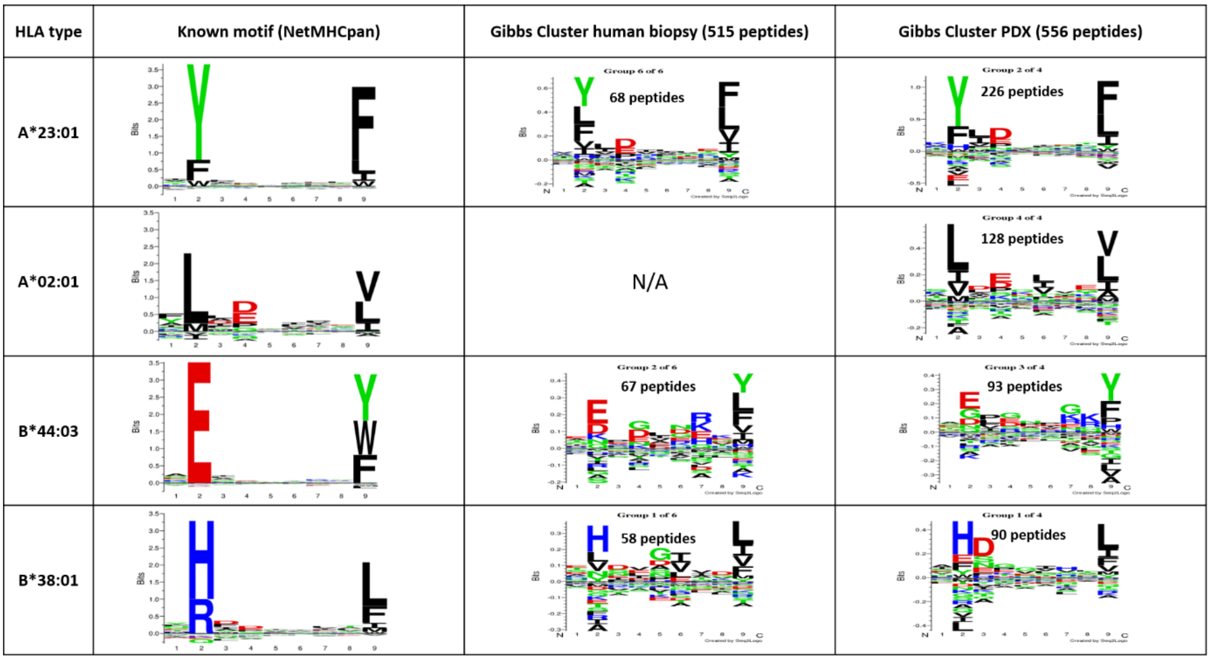

Figure S4: Gibbs cluster of the HLA peptides from patient P4 biopsy and PDX tumor in comparison to their known sequence motifs. The known consensus sequence motifs of the HLA allotypes of Patient 4 (gastric carcinoma) were taken from NetMHCpan and displayed next to the patient's Gibbs clustering of his biopsy and PDX tumor. The number of peptides used for each cluster is indicated above the logo.

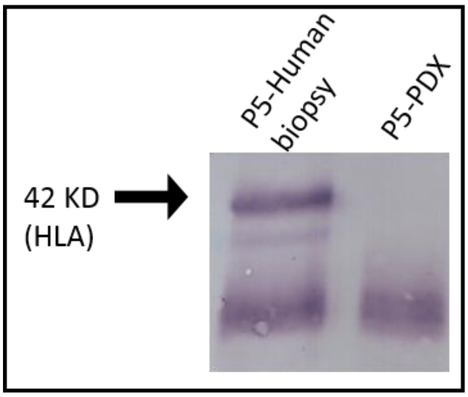

Figure S5: Western blot showing the absence of the HLA from the PDX sample of patient P5 (vascular hemangioendothelioma). Lack of the HLA heavy chain (42 kD band) correlates with the smaller number of human HLA peptides detected in the PDX tumor sample, compared to the patient biopsy.

Western blot method

Before MS/MS analysis, a fraction of the HLA molecule sample was subjected to western blot analysis to validate the immuno-purification process. Protein samples, resolved by SDS-PAGE gel, were electro-transferred to a nitrocellulose membrane (90 V, 1 h, on ice). The membrane was then blocked with 10% skimmed milk in PBS supplemented with 1% Tween PBST (1 h, room temperature [RT]). Then, the membrane was washed three times with 0.3% PBST and incubated (1 h, RT) with primary monoclonal rabbit anti-human HLA-A class I antibody (ab52922, Abcam, Cambridge, United Kingdom), diluted (1:5000) in 0.3% PBST. Then, the membrane was incubated (1 h, RT) with secondary polyclonal alkaline phosphatase goat anti-rabbit antibody (A9919, Sigma), diluted in 0.3% PBST. The membrane was then washed three times with 0.3% PBST and one time with alkaline phosphate (AP) buffer, followed by development of the color signal with a solution of 0.33 µL of 50 mg/ml BCIP (5-Bromo-4-chloro-3-indolyl phosphate) and 0.66 µL of 50 mg/ml NBT (nitro blue tetrazolium chloride), in 10 ml AP buffer.

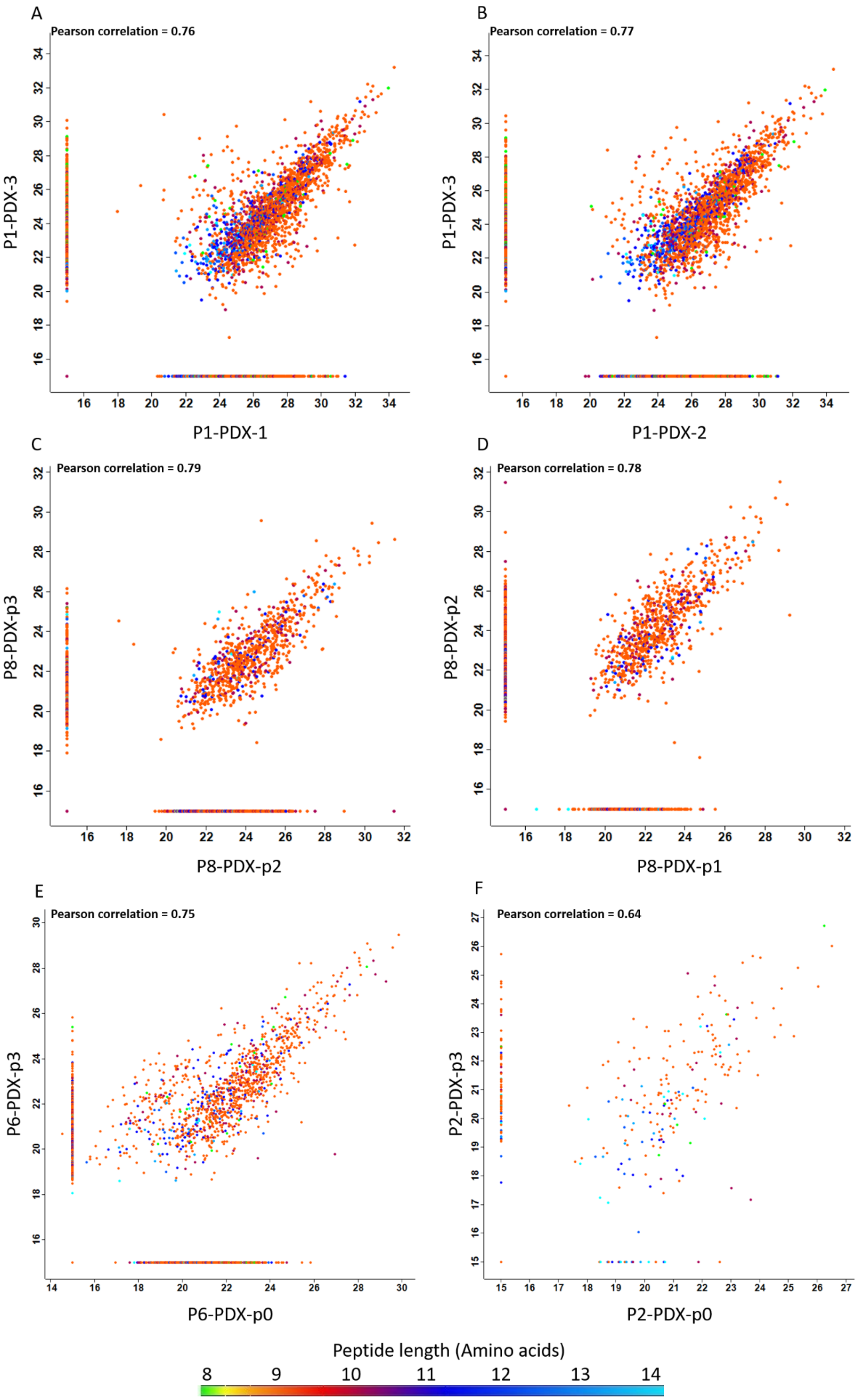
A
Pearson correlation = 0.76
P1-PDX-3
P1-PDX-1
B
Pearson correlation = 0.77
P1-PDX-3
P1-PDX-2
C
Pearson correlation = 0.79
P8-PDX-p3
P8-PDX-p2
D
Pearson correlation = 0.78
P8-PDX-p2
P8-PDX-p1
E
Pearson correlation = 0.75
P6-PDX-p3
P6-PDX-p0
F
Pearson correlation = 0.64
P2-PDX-p3
P2-PDX-p0
Peptide length (Amino acids)
8 9 10 11 12 13 14

Figure S6: Similar HLA peptidomes were detected in different PDX tumors originating from the same patient. Each dot represents the relative LC-MS signal intensity of a single peptide, on a log2 scale, with the color indicating the peptide length, as per the scale at the bottom. The group of HLA peptides detected in only one of the samples is indicated on the vertical/horizontal lines with the imputed arbitrary numbers of 15. A & B) Same passage PDX tumors from head and neck adnexal adenocarcinoma (patient P1).C & D) Different passage PDX tumors from pancreatic adenocarcinoma (patient P8). E) Different passage PDX tumors from head and neck squamous cell carcinoma (patient P6) F) Different passage PDX tumors from bile duct cholangiocarcinoma (patient P2).

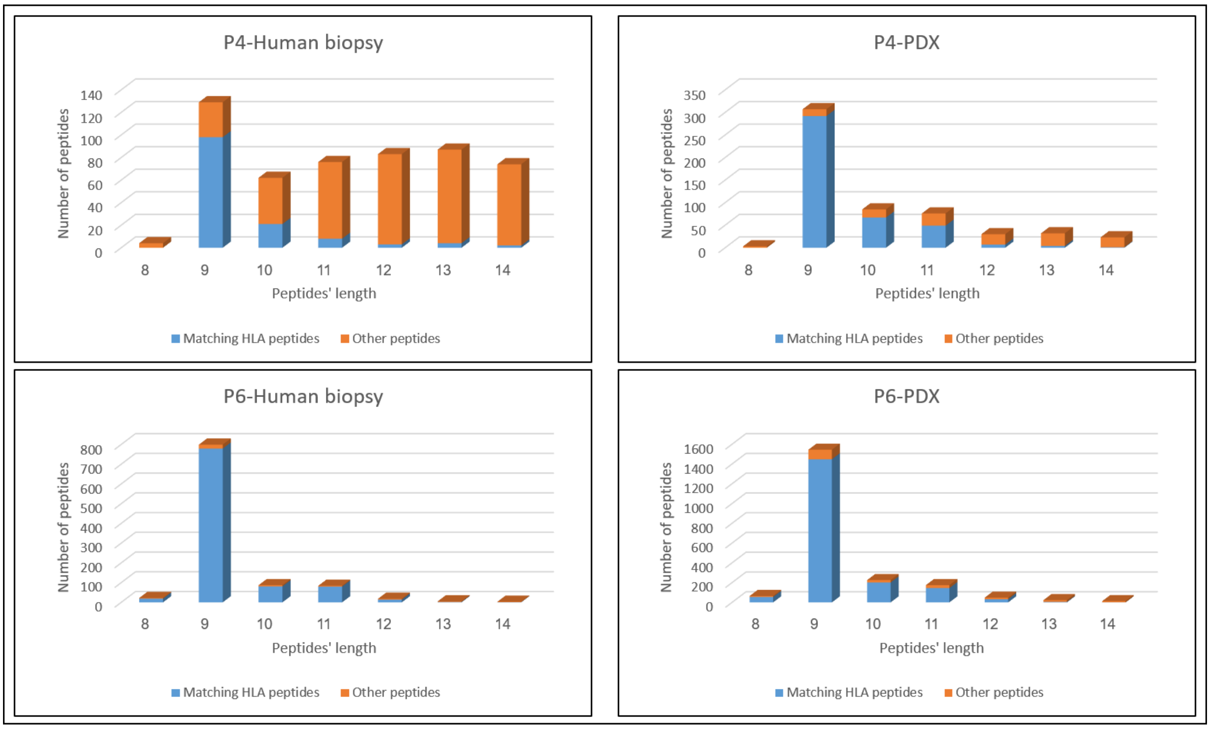

Figure S7: The length distribution of the peptides provides an indication for the percentage of true HLA ligands. Samples are of patient P4 (gastric carcinoma, top row) and patient P6 (head and neck squamous cell carcinoma, bottom row). Color indicates fitness to the HLA consensus sequence motifs of the patients.

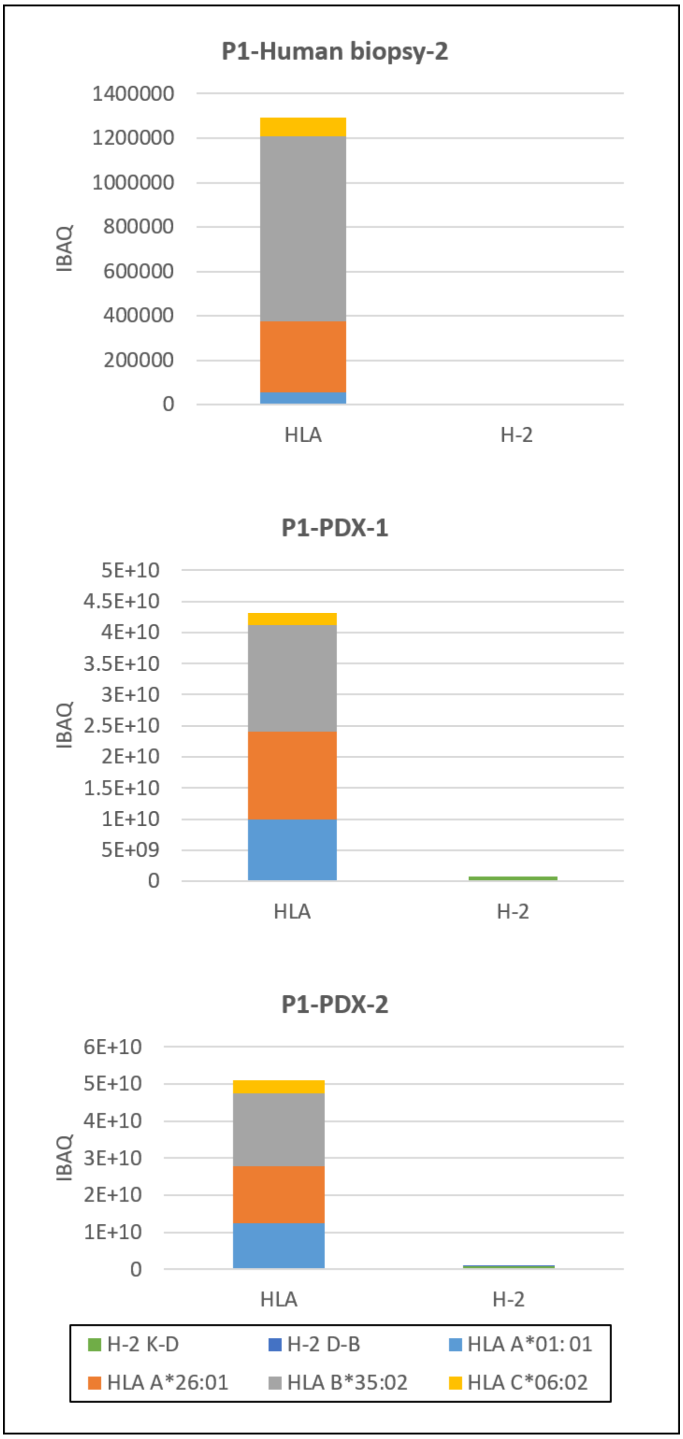

Figure S8: Relative amounts (in iBAQ values) of the different MHC allomorphs recovered from the immunoaffinity columns of PDX tumor and human biopsy specimens of patient P1.

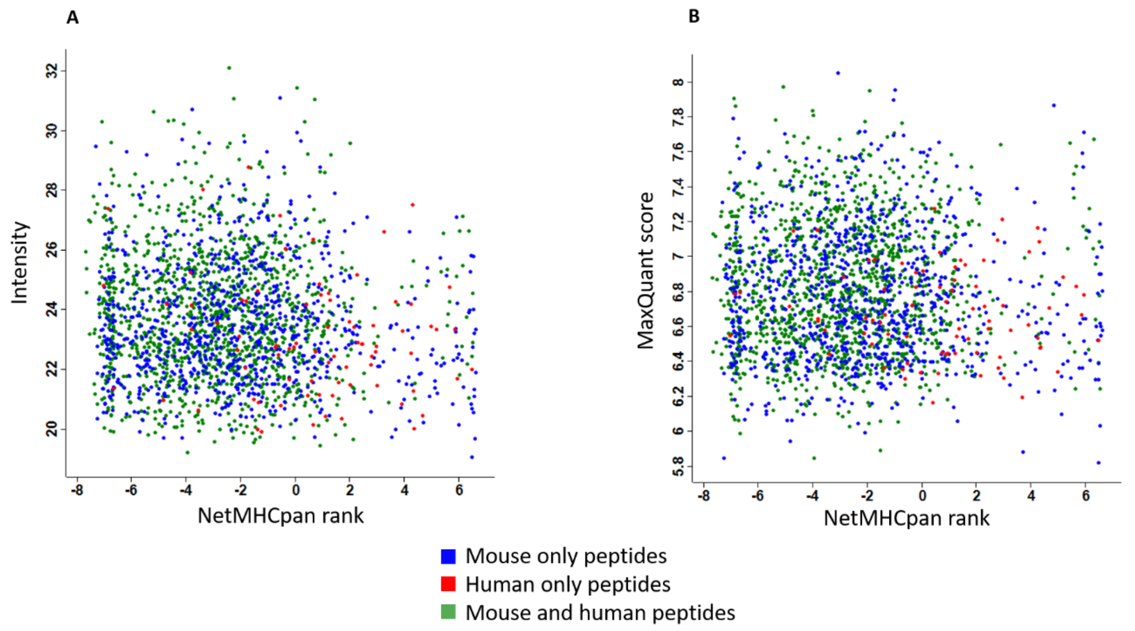

Figure S9: The intensities and MaxQuant scores and NetMHCpan ranks of mouse peptides are distributed randomly, like the human peptides, in patient P6 (head and neck squamous cell carcinoma). Each dot represents a single peptide, on a log2 scale of the intensities/MaxQuant scores relative to the NetMHCpan rank, with the color indicating the protein origin-legend at the bottom. A) intensity vs. NetMHCpan rank B) MaxQuant score vs. NetMHCpan rank.

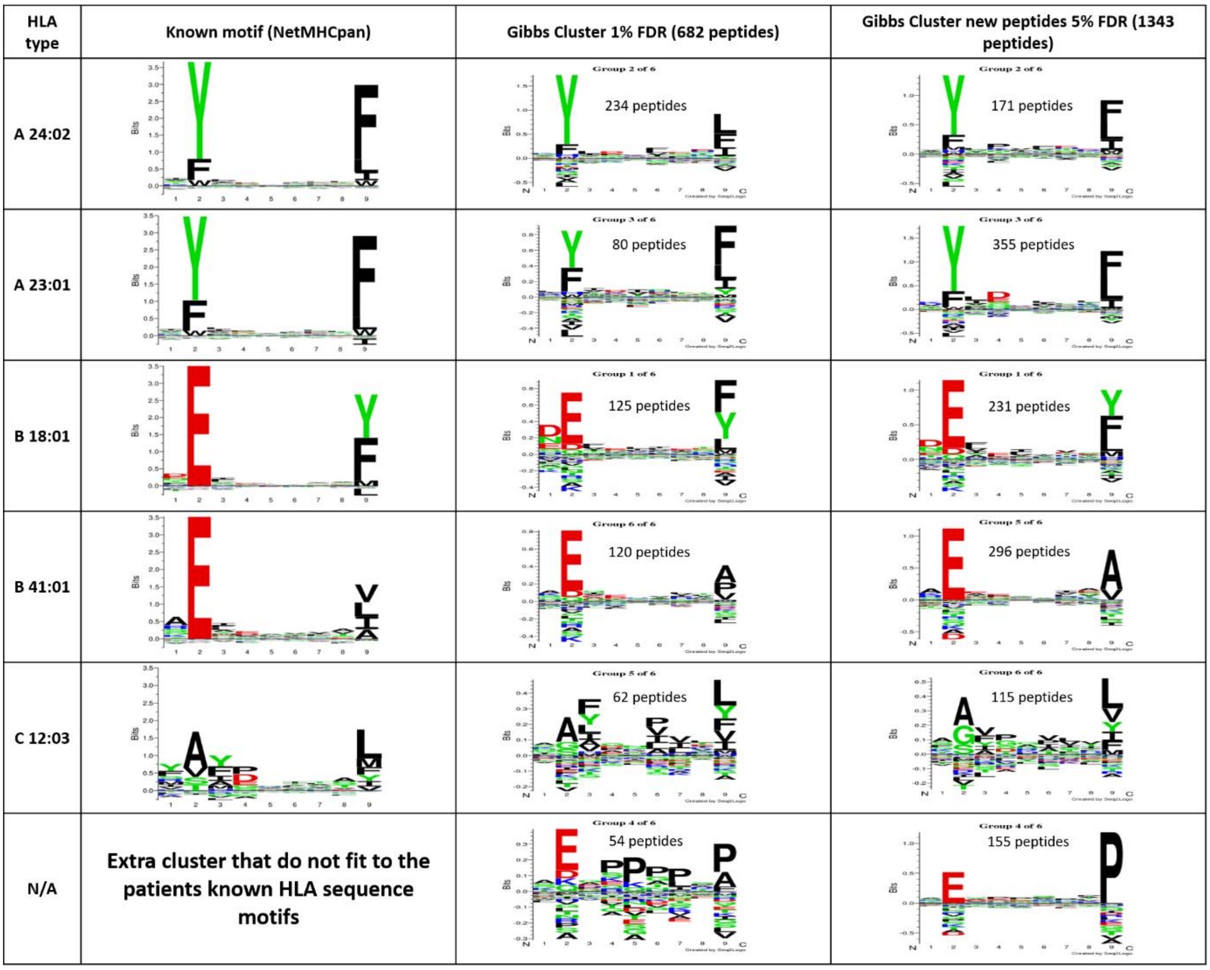

Figure S10: Gibbs cluster of the HLA peptides from patient P6 detected in 1% FDR analysis in comparison to the new identified peptides added by the 5% FDR analysis and to their known sequence motifs. The known consensus sequence motifs of the HLA allotypes of Patient 6 (head and neck squamous cell carcinoma) were taken from NetMHCpan and displayed next to the 1% FDR and newly identified peptides in 5% FDR analysis, Gibbs clustering. The numbers of peptides used for each cluster is indicated above the logo.

Supplemental table:

Table S1: Summary of patient HLA allotypes

patient	Locus	HLA-Allele 1	HLA-Allele 2
P1	A	A*01:01	A*26:01
	B	B*35:02	B*37:01
	C	C*06:02	C*06:02
P2	A	A*24:02	A*24:02
	B	B*58:01	B*58:01
	C	C*07:01	C*07:01
P3	A	A*02:01	A*24:02
	B	B*38:01	B*51:01
	C	C*12:03	C*14:02
P4	A	A*02:01	A*23:01
	B	B*38:01	B*44:03
	C	C*04:01	C*12:03
P5	A	A*29:02	A*31:01
	B	B*14:02	B*35:02
	C	C*04:01	C*08:02
P6	A	A*23:01	A*24:02
	B	B*18:01	B*41:01
	C	C*12:03	C*17:01
P7	A	A*25:01	A*25:01
	B	B*18:01	B*38:01
	C	C*12:03	C*12:03
P8	A	A*26:01	A*29:02
	B	B*38:01	B*44:03
	C	C*12:03	C*16:01
